# Laryngeal Papillomatosis in Adults: Assessment for Ten Years at the ENT Department of the National University Hospital of Fann (Dakar, Senegal)

**DOI:** 10.1155/2020/2782396

**Published:** 2020-08-03

**Authors:** N. Ndour, S. Maiga, A. Houra, R. E. A. Deguenonvo, C. Ndiaye, N. Pilor, A. Mbaye, A. C. Sall, M. S. Diouf, I. C. Ndiaye

**Affiliations:** ^1^ENT Department of the National University Hospital (NUH) of Fann, Dakar, Senegal; ^2^ENT Department of Idrissa Pouye Hospital, Dakar, Senegal

## Abstract

**Objectives:**

The aim of this study was to describe the epidemiological, diagnostic, and therapeutic aspects of adult laryngeal papillomatosis in Senegal. *Patients and Methods*. This is a retrospective descriptive study of patients aged above 18 years with laryngeal papillomatosis and followed at the ENT department of the NUH of Fann between 01 January 2009 and 31 December 2018.

**Results:**

The mean age at diagnosis was 37.74 years and a sex ratio of 0.93. The 20–29 age group was the most represented (45.2%). The average consultation delay was 8.34 years. All patients had dysphonia at the moment of the diagnostic and in 35.5% of cases, and it was associated with laryngeal dyspnea. Glottis localization was present in all our patients, i.e., 100% of the cases. A tracheotomy was performed in 9.67% of cases. All of our patients have had their papilloma peeled per endoscopic with tweezers. No cases of malignant degeneration were found in our study.

**Conclusion:**

Laryngeal papillomatosis is the most common benign tumor of the larynx in both children and adults. Despite the progress of endoscopy and antiviral treatments, its treatment poses many problems in our undermedicalized countries.

## 1. Introduction

As early as the 17th century, Marcellus Donnalus described these “warts in the throat;” however, it was not until 1871 that the term papillomatosis was given to these lesions by Sir Morell Mackenzie. Laryngeal papillomatosis is therefore a benign, squamous tumor proliferation developed at the laryngeal mucosa. It is a recurrent disease of viral origin (human papillomavirus types 6 and 11) that can affect the entire respiratory tract and the upper digestive area [1–3]. This condition affects both children and adults. Although it is histologically benign, it is recognized as having a potential for malignant degeneration that should not be overlooked [4]. However, recidivism is the predominant event in its evolution. In addition, laryngeal papillomatosis when obstructive is life-threatening, making a rescue tracheotomy indicated [5].

Despite the extensive knowledge acquired about its clinical and histological features, light remains to be shed on its etiopathogenesis and therapy [2, 4].

Our objective was therefore to study adult laryngeal papillomatosis in the ENT and neck and face surgery department of the NUH of FANN in Dakar, Senegal, between January 1, 2009, and December 31, 2018, in order to analyze its epidemiological, clinical, and therapeutic aspects.

## 2. Patients and Methods

This retrospective study concerns 31 cases of laryngeal papillomatosis in adults collected over a period of 10 years (from January 1, 2009, to December 31, 2018), in Lamine Sane Diop University ENT department of the NUH of Fann in Dakar.

We have excluded from this work all records of patients under the age of 18. The following parameters were studied: sex, age, time of consultation, reasons for consultation, location of lesions, treatments performed, histological results, and evolution after treatment.

The data obtained were entered into Excel and analyzed by the statistical package for social science (SPSS version 20).

## 3. Results

### 3.1. Epidemiological Aspects

**Figure 1:**
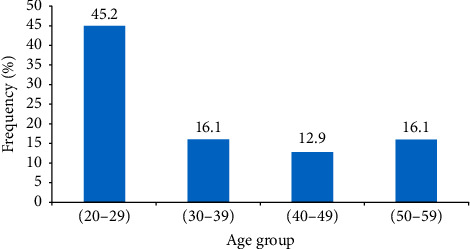
Distribution of patients by the age group.

Over a 10-year period, we collected 31 cases of adult laryngeal papillomatosis, an incidence of 3.1 new cases/year. This study population consisted of 15 males and 16 females, for a sex ratio of 0.93. The average age was 37.74 years with extremes ranging from 20 to 72 years. The median was 30 years, the standard deviation was 14.5, and the age range of 20 to 29 years was the most represented with 14 cases (Figure 1).

Four of our patients had been treated for laryngeal papillomatosis during a progressive pregnancy, i.e., 12.9% of cases. In our series, 15 patients or 48.4% of the cases had already received previous tweezers peeling treatment in another ENT department of the place.

### 3.2. Diagnostic Aspects

The average time between the onset of the first symptoms and the first consultation was 100.16 months (8.34 years) with extremes ranging from four months to 360 months (30 years).

The functional symptomatology was dominated by chronic dysphonia, which was found in all our patients. It was an isolated dysphonia, and in 35.5%, it was associated with laryngeal dyspnea. Other functional signs such as swallowing discomfort, coughing, and respiratory sounds were absent in all patients.

**Figure 2:**
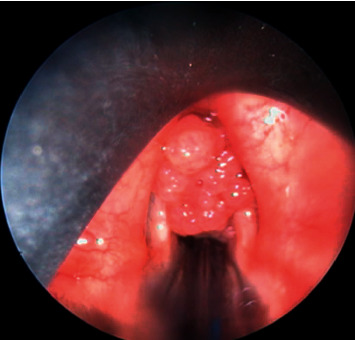
Endoscopic image showing clusters of papilloma.

Examination of the larynx allowed the identification of papilloma and preserved bilateral laryngeal mobility. The endoscopy under general anesthesia performed in all our patients visualized the papilloma as raspberry-, pinkish-, and greyish-looking lesions in clusters (Figure 2).

The glottis area was reached in all cases (31 patients) at the first endoscopy. The glottis and the subglottis localizations were found in 29% of cases and the involvement of the three stages of the larynx in 12.9% of cases. Pure glottis localization was noted in 58.1%.

Only one patient had an extralaryngeal location, as tufts of papilloma were found on the uvula of a 40-year-old pregnant woman.

### 3.3. Therapeutic Aspects

Emergency tracheotomy was performed in 3 patients in our study, i.e., 9.67% of cases. In two cases, the tracheotomy was performed under local anesthesia for severe laryngeal dyspnea and in one patient following direct suspension laryngoscopy.

Endoscopic papilloma peeling with tweezers under direct laryngoscopy was performed in all our patients. The number of endoscopies per patient averaged 2 sessions with extremes ranging from one to fourteen (14) sessions.

A short ten (10) days period of corticosteroid therapy was routinely initiated in all our patients.

Vaporization of papilloma with CO_2_ laser was performed in 5 patients following recurrences after several peeling sessions. One session was conducted for each of the three patients and two sessions each for the others.

None of our patients had received antiviral treatment.

### 3.4. Histological Aspects

The results of the anatomopathological examination were only available in 24 patients or 77.4% of the cases with a confirmed diagnosis. Among these patients, ten (10) or 41.7% of the cases presented dysplasia of different grades without identified malignant degeneration.

### 3.5. Evolutionary Aspects

**Figure 3:**
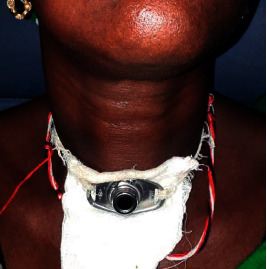
Tracheotomy cannula in a patient with recurrent laryngeal papillomatosis.

Of the three tracheotomized patients, two had their cannula taken off after 48 hours. About the third patient still carrying the tracheotomy cannula (Figure 3), a nasofibroscopic examination revealed anterior glottis stenosis after 14 sessions of endoscopic peeling. We registered an improvement in voice in 20 patients, i.e., 64.5% of cases, and complete remission in 4 patients, including one of the patients who had received LASER spray. A recurrence was detected in 19 patients, i.e., 61.2% of cases with a mean time to recurrence of 9 months. In four of these patients, a LASER spray was used. Twelve patients were lost to follow-up after their first endoscopy. One death by asphyxia was reported in a patient for whom the tracheotomy could not be performed in time in the event of a recurrence.

## 4. Discussion

Laryngeal papillomatosis is a rare condition in Africa but even more so in the rest of the world [2]. In the United States, Derkay reported an annual incidence of 1.8 cases per 100,000 adults [6]. Cristensen et al. [7] reported 0.2–0.7 cases per 100,000 inhabitants in Sweden in 1984. In Africa, a variable annual frequency of 3.5 to 5 cases/year has been reported in the series of Sereme et al. in Cameroon, Timbo et al. in Mali, and Pegbessou et al. in Togo [8–10]. In our series, we have identified an annual incidence of 3.1 cases/year, which corroborates our department's previous data [3, 5].

It is a benign tumor mainly in children, but it is well represented in the adult population with a peak frequency in the 20- to 30-year age group found in some series [2, 10, 11]. Our study finds a median age of 30 years with extremes ranging from 20 to 72 years, with a slight predominance of females, which is consistent with the results of the literature [9, 11].

In the literature, laryngeal papillomatosis is often diagnosed late [3, 5, 12]. In our study, from the appearance of the first signs to the first consultation, we observe an average duration of 8 years 4 months with extremes ranging from 4 months to 30 years. In our context, one could attribute this delay of consultation to the low socioeconomic level and the difficulty in access to care due to the lack of ENT practitioners in most regions of Senegal. In addition, dysphonia in adults was often trivialized by the patient, and dyspnea occurred rarely and after a long period of disease progression.

In adults, dysphonia is the main telltale sign of the disease. It is a chronic, permanent, progressively worsening dysphonia, signaling the organic character [3, 5, 13]. It was present in all our patients and was isolated in 64.5% of cases. These results are superior to those of several series in which it was found in 41%, 48%, and 49% of cases, respectively [5, 8, 10]. This difference could be due to the fact that our study only involved adults, unlike the others. Apart from the narrower larynx of the child, laryngeal papillomatosis often goes unnoticed in the dysphonia phase and is only discovered in the course of laryngeal dyspnea [2].

Laryngeal dyspnea of varying severity is rare in adult laryngeal papillomatosis, and unlike in children, it can cause respiratory distress requiring emergency tracheotomy [5, 12, 13]. In our series, it was found in 35.5% of our patients, in correlation with the results of Pegbessou et al. (31%) [10] and Sereme et al. (45%) [8]. This can be explained by the anatomical peculiarities of the larynx in children: an edema of 1 mm thickness will reduce the subglottic duct by 50%, whereas in adults, an edema of 2.5 mm would be needed to obtain the same result [14].

Examination of the larynx using indirect laryngoscopy or nasofibroscopy allows visualization of the papillomas and assessment of the laryngeal mobility that is generally preserved [3, 5]. It was practiced in 28 patients, i.e., 90.32% of cases.

The examination under general anesthesia allows a macroscopic diagnosis and a lesion assessment to be made. It can be used to visualize raspberry-, pink-, and grayish-looking lesions in clusters [3, 12]. These lesions usually begin at the floor of the ventricular bands, at the anterior commissure, on the anterior third of the vocal cords, and may extend to the entire larynx, or even the hypopharynx and the entire respiratory tract [3, 12]. In our series, the glottis was affected in all of our patients, which corroborates the results in the literature [3, 5, 9, 12]. This may explain the frequency of dysphonia. Only one extralaryngeal location was found, a uvula involvement in a 40-year-old pregnant woman.  Table 1 summarizes the frequency of other locations in selected series.

During endoscopy, samples are systematically taken for histological examination to confirm the diagnosis but also to look for malignant transformation, especially in adults.

Laryngeal papillomatosis is a recurrent condition that is currently treated primarily symptomatically [3, 12, 13]. Several treatment strategies have been developed, but none of them have been proven effective. The challenge is to maintain airway open and improve voice quality [3].

In developing countries such as ours, treatment options are limited to per endoscopic excision with forceps. All the patients in our study had their papillomas peeled by endoscopic tweezers, in accordance with numerous authors [3, 5, 8, 9, 12]. The number of endoscopy sessions ranged from one to fourteen sessions with an average of 2 sessions per patient. In young subjects, repeated monthly endoscopy and peeling sessions are needed during the first years [2].

In our context, we find it very difficult to repeat the endoscopy because of the cost of it, but most of our patients are lost from sight at the first endoscopy.

The tracheotomy is life-saving in our context, showing a delay in consultation, by Ndiaye et al. in 50.8% of cases, Maiga et al. in 20.8% of cases, and Maliki et al. in 76% of cases. In our series, the tracheotomy was performed in only 9.67% of cases. This is explained by the fact that our study only concerns adult subjects.

LASER vaporization is an interesting therapeutic alternative in the management of laryngeal papillomatosis. It is performed under general anesthesia in per endoscopic surgery [15]. The study by Derkay [6] showed that 92% of American ENTs who treated papillomatosis used CO_2_ laser. However, in our study, five patients had benefited from this type of treatment within the framework of humanitarian missions because our structure does not have LASER.

The microdebrider [16, 17], like the CO_2_ laser, is one of the new therapies used in the management of laryngeal papillomatosis. None of the patients in our series benefited from it due to the lack of an adequate technical equipment.

Since 1998, intralesional injection of cidofovir after surgical removal of papilloma has been an alternative treatment in cases of recurrence [18]. However, it remains financially inaccessible, and no patients in our series have benefited from it.

An anatomopathological study is necessary especially in adults where a malignant transformation is to be feared. The link between papillomatosis and the occurrence of epithelioma is not easily established with certainty. The diagnosis is suspected in cases of rapid spread or bleeding [19, 20].

In our series, we found 41.7% dysplasia; however, no cases of malignant degeneration were found.

## 5. Conclusion

Laryngeal papillomatosis is the most common benign tumor of the larynx in both children and adults. Despite the progress of endoscopy and antiviral treatments, its management poses many problems in our undermedicalized countries.

## Figures and Tables

**Table 1:** Different locations of papillomas according to the authors.

Authors (years)/Reference	Number of cases	Subglottis	Glottis	Subglottis	Trachea
Timbo et al. (2002) [9]	19	2	19	4	4
Malick et al. (2008) [5]	61	13	61	253	1
Pegbessou (2011) [10]	39	4	35	3	0
Maliki et al. (2012) [12]	21	3	21	6	0
Maiga et al. (2017) [3]	48	17	48	17	0
Our study (2019)	31	9	13	4	0

## Data Availability

The data used to support the findings of this study (Excel database) are available from the corresponding author upon request.
